# Motor and functional recovery after stroke: a comparison between rehabilitation settings in a developed versus a developing country

**DOI:** 10.1186/1472-6963-14-82

**Published:** 2014-02-22

**Authors:** Anthea Rhoda, Mario Smith, Koen Putman, Ratie Mpofu, Willy DeWeerdt, Liesbet DeWit

**Affiliations:** 1 Faculty of Community and Health Sciences, University of the Western Cape, Private Bag X17, 7535 Bellville, Western Cape, South Africa; 2 Medical Sociology, Faculty of Medicine and Pharmacy, Vrije Universiteit, Brussel, Laarbeeklaan 103, 1090 Jette, Belgium; 3 Faculty of Kinesiology and Rehabilitation Sciences, Eekenhoornlaan 34, B-3210 Linden, Belgium

**Keywords:** Stroke, Recovery, Rehabilitation, Developing countries, Developed countries

## Abstract

**Background:**

Recovery post stroke is well documented in the field of stroke rehabilitation. The structure and process of rehabilitation are different between developed and developing countries. The aim of the present study was to compare the motor and functional recovery of stroke patients in Germany versus stroke patients receiving rehabilitation in South Africa.

**Methods:**

This study used secondary data analysis of patient protocols collected in two independent studies conducted in Germany and South Africa respectively. A total of 73 patients from the two separate studies were matched for age at stroke onset, gender, and initial motor functioning. Motor and functional recovery were assessed at baseline, two and six months post stroke using the Rivermead Motor Assessment Scale and the Barthel Index (BI) respectively. Significant differences in motor and functional recovery were found, using the Wilcoxon rank sum test on admission to the centre, and at two and six months after stroke. A generalized linear mixed-methods model (GLIMMIX) was used to compare the recovery patterns between the participants from the two settings over time.

**Results:**

The results of the GLIMMIX revealed a significant difference in favour of the German participants for gross motor (RMA-GF) and upper limb (RMA-A) recovery, while no significant difference was found for lower limb (RMA-LT) and functional (BI) recovery patterns between the participants of the two settings. No significant differences existed in RMA-A and BI-scores on admission to the CHC/SRU. At two and six months after stroke, both the RMA-A and BI-scores were significantly lower in the South African than the German sample.

**Conclusion:**

The results of this study provide empirical evidence for differential recovery patterns for patients in developed and developing countries. A detailed exploration of the factors to which this difference in recovery patterns can be attributed was beyond the scope of the present study, and is recommended for future research.

## Background

Facilitating recovery post stroke is an important goal of rehabilitation [1,2]. The recovery patterns over time and outcomes at specific time points are variables which are often investigated in research into stroke rehabilitation [3-5]. In addition research in this field also investigates variables such as process of rehabilitation [4] and the content and intensity of rehabilitation [5,6]. These variables have been investigated in a number of different settings and contexts where the rehabilitation approaches could differ.

The body of literature on stroke rehabilitation clearly establishes that there are different approaches to stroke rehabilitation in developed [4,5] and developing countries [7-10]. The differential approaches do not necessarily represent preferred models or best practice. Although rehabilitation in developed countries tends to follow recommended stroke rehabilitation guidelines [9,11], in developing countries rehabilitation provided is often dependent on the availability of resources, which are often limited [10].

The majority of studies investigating recovery and outcome after stroke have been conducted in developed high-income countries [4,5,12]. It emerges that admission to in-patient rehabilitation facilities is the norm in developed countries [9,13] where factors that enhance the outcome of rehabilitation are more readily available. In contrast, only limited literature is available that reports empirically on outcomes and recovery patterns of stroke patients living in developing or under-resourced countries such as Africa, including South Africa. The lack of resources often results in the lack of adherence to global best practice guidelines, which could influence the outcomes and recovery post stroke. Rehabilitation at out-patient facilities is more common in developing countries. Also a multidisciplinary approach is seldom applied because some disciplines are not being employed at the centres [14].

The differences in stroke rehabilitation between developed and developing countries are readily acknowledged in common perception, but are not empirically supported. Research into rehabilitation has maintained the binary construct of stroke rehabilitation, by focusing separately on treatment aspects in developed and developing countries. The typical outcomes and recovery patterns across both types of country are seldom directly compared. According to our knowledge, there is no documented information comparing the outcomes of stroke rehabilitation in developed (well-resourced) and developing (low-resourced) countries.

The aim of the present study is to compare motor and functional recovery patterns, as well as functional outcomes, in stroke patients receiving rehabilitation in different treatment regimes relating to the process of rehabilitation in centres from developed and developing countries. This comparison could provide empirical support for the perceived differences in the recovery and outcomes of stroke patients living in developed and developing countries.

## Methods

### Patients and settings

Patients were included from two previous studies, which represent stroke rehabilitation in developing and developed countries. Out-patient rehabilitation offered at Community Health Centres in South Africa is an example of rehabilitation in developing countries, and an in-patient rehabilitation centre in Germany is an example of rehabilitation in developed countries. The primary motivation for selecting these settings is that they represent the typical practice model in developing and developed countries respectively.

**Figure 1:**
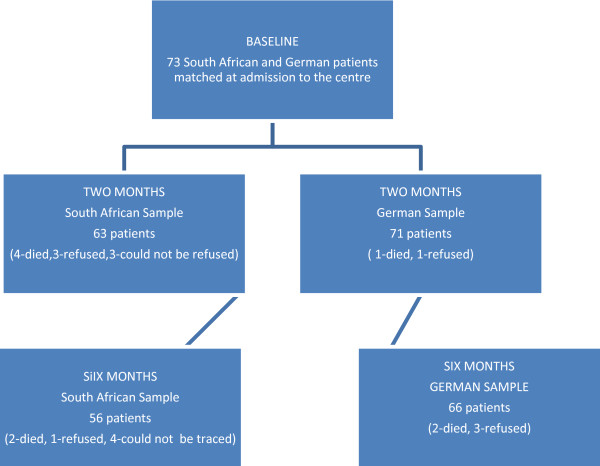
Illustrates the recruitment and lost-to-follow-up of the participants at the different assessment points.

The German cohort was selected from the CERISE study (Collaborative Evaluation of Rehabilitation in Stroke across Europe) that compared stroke care and recovery patterns in four European rehabilitation centres [4]. In-patient multidisciplinary care was provided in a stroke rehabilitation unit (SRU). Patients were recruited consecutively, using the following inclusion criteria: first-ever stroke as defined by WHO [15], age 40 to 85 years, and Rivermead Motor Assessment scores; [16] gross function (RMA-GF) ≤ 11, and/or leg and trunk function (RMA-LT) ≤ 8; and/or arm function (RMA-A) ≤12 on admission to the centre. Exclusion criteria were: other neurological impairments with permanent damage; stroke-like symptoms attributable to subdural hematoma, tumour, encephalitis or trauma; admission to the centre > 6 weeks after stroke; no informed consent; and pre-stroke Barthel Index [17] <50. The sample in this study comprised 135 patients. Figure 1 illustrates that in the German (GE) sample, two patients were lost-to-follow-up at two months (one died and one refused) and another five at six months after stroke (two died and three refused).

The South African study (SA) [18] aimed at documenting the recovery patterns of 100 patients with stroke who were admitted to 21 Community Health Centres (CHCs) in the Cape Town Metropolitan district of the Western Cape Province. Physiotherapy services were provided at all the CHCs (n = 21), while occupational therapy and speech therapy services were provided in 16 and two CHCs respectively. Patients were recruited consecutively from the 21 CHSs using identical criteria for inclusion and exclusion to those in the CERISE study, except for age. In South Africa, stroke occurs in a younger population [14], therefore the age range was widened to 35–85 years in order to include a representative patient sample in the South African CHC study. In this sample, 10 patients were lost-to-follow-up at two months (four died, three refused and three could not be traced), and another seven patients were lost at six months after stroke (two died, one refused and four could not be traced) see Figure 1.

For inclusion in the present study, matched patient pairs were identified from the South African and German groups. Patients were matched on age at stroke onset (plus/minus 5 years), gender, and RMA-GF score (plus/minus 1 point) on admission. The final sample comprised 73 matched pairs.

### Participant assessment

On admission to the SRU or CHC, patients’ age, gender, urinary incontinence (defined as a score <10 on item ‘bladder’ of the BI), aphasia (defined as a score >0 on item 9 of the National Institute of Health Stroke Scale, (NIHSS) [19] (and dysarthria (defined as a score >0 on item 10 of the NIHSS) were assessed. In addition, motor and functional recovery was assessed at admission to the SRU or CHC, and at two and six months after stroke onset, using the Rivermead Motor Assessment [16] (sections; gross motor function = RMA-GF, leg and trunk = RMA-LT and arm = RMA-A) and the Barthel Index [19] respectively. A six-month follow-up period was used because the majority of motor recovery occurs before that time point [20]. A researcher in the European centre collected the data. At the start of the study, the researcher was trained in the assessments during a workshop. A manual was provided to ensure standardization. The project manager (LDW) visited the centre four times to recalibrate the researchers’ work. The same training and recalibration method was provided to the South African researcher (AR).

### Statistical analysis

Demographic and clinical data of both matched patient samples was presented in terms of means with standard deviations, medians with interquartile ranges, or frequencies with percentages, as appropriate. The Mc-Nemar test was used to compare the prevalence of urinary incontinence, aphasia and dysarthria with both matched samples, while the T-test for dependent samples was used to test for significant differences based on age.

The Wilcoxon rank sum test was used to assess significant differences in motor and functional ability (scores) between the South African and the German patient sample at admission to the centre, two and six months after stroke. In addition, motor and functional recovery patterns were compared between both patient samples, using a generalized linear mixed methods model (GLIMMIX). GLIMMIXs are mixed models that can be used with discrete outcomes. Such models correct for the correlation between repeated observations with subjects. They also provide valid inferences for missing observations, provided that their absence does not depend on unobserved outcomes (i.e. assuming missingness at random) [21]. It should be further noted that GLIMMIX models compare the steepness of the recovery slope between the two groups taking into account the longitudinal study design, whereas the Wilcoxon rank sum test only compares outcomes at a certain point in time, not taking into account the patients’ initial scores.

The models were fitted with the GLIMMIX procedure. All statistical analyses were performed using SAS, version 9.2, and tested for significance at a 0.05 alpha level (p < 0.05).

### Ethics

Ethical clearance for the South African study was obtained from the University of the Western Cape’s Senate Ethics Committee and for the German study from the ethics committee of the German Rehabilitation Centre where the study was conducted.

## Results

### Participants

Demographic and clinical characteristics were presented and compared between both samples (Table 1 and 2). No significant differences existed for age, gender and aphasia on admission. Urinary incontinence (p = 0.03) and dysarthria (p = 0.02) occurred significantly more in the German than in the South African sample.

**Table 1:** Comparisons of clinical data between matched South-African (SA) (n = 73) and German (GE) patient samples (n = 73)

**Parameters**	**SA n = 73**	**GE n = 73**	**p-value**
Age in years: mean (SD)	63.4 (10.0)	63.9 (9.2)	0.15^a^
Gender: male: n (%)	28 (38.4)	28 (38.4)	1.00^b^
Female: n (%)	45 (61.6)	45 (61.6)	
Urinary incontinence: n (%)	11 (15.1)	22 (30.1)	**0.03**^**b**^
Dysarthria: n (%)	25 (34.2)	39 (53.4)	**0.02**^**b**^
Aphasia: n (%)	15 (20.5)	20 (27.4)	0.35^b^
TSO median days (q1 – q3)	21 (15–31)	20 (16–27)	0.32^c^

^a^paired T-test, ^b^Mc-Nemar test, ^c^Wilcoxon signed rank test, TSO: time since stroke onset, IQR: Interquartile range, p-values < 0.05 are in bold.

**Table 2:** Comparisons of admission, two and six months post stroke data between matched South-African (SA) (n = 73) and German (GE) patient samples (n = 73)

**Parameters**	**SA**	**GE**	**p-value**
	**n = 73**	**n = 73**	
RMA-GF			
On admission to CHC/SRU: median (IQR)	8 (4–11)	8 (4–10)	0.28^c^
At two months after stroke: median (IQR)	11 (6–11)	10 (6–11)	**0.03**^**c**^
At six months after stroke: median (IQR)	11 (8–11)	11 (9–12)	**0.03**^**c**^
RMA-LT			
On admission to CHC/SRU: median (IQR)	5 (3–7)	7 (5–8)	**0.0001**^**c**^
At two months after stroke: median (IQR)	7 (3–8)	9 (7–9)	**<0.0001**^**c**^
At six months after stroke: median (IQR)	7 (5–9)	9 (7–10)	**<0.0001**^**c**^
RMA-A			
On admission to CHC/SRU: median (IQR)	4 (1–9)	6 (1–10)	0.09^c^
At two months after stroke: median (IQR)	8 (2–11)	9 (3–14)	**0.03**^**c**^
At six months after stroke: median (IQR)	8 (1.5-11)	12 (5–14)	**0.0003**^**c**^
BI			
On admission to CHC/SRU: median (IQR)	65 (50–80)	80 (45–90)	0.05^c^
At six months after stroke: median (IQR)	85 (65–95)	95 (80–100)	**0.003**^**c**^

^c^Wilcoxon signed rank test.

SA indicates South-African patient sample, GE: German patient sample; CHC: Community Health Centre; SRU: Stroke Rehabilitation Unit; RMA-GF: Rivermead Motor Assessment- Gross Function, RMA-LT: Rivermead Motor Assessment- Leg and Trunk; RMA-A: Rivermead Motor Assessment Arm Function, BI: Barthel Index, IQR: Interquartile range, p-values < 0.05 are in bold.

### Motor and functional outcome

On admission to the CHC/SRU, RMA-GF scores did not differ significantly between the two samples. At two and six months after stroke, RMA-GF scores differed significantly between patient samples, with higher scores for the South African patients at two months after stroke, and higher scores for the German patients at six months after stroke. On admission to the CHC/SRU, at two and six months after stroke, the RMA-LT scores were significantly lower in the South African than in the German sample. No significant differences existed in RMA-A and BI-scores on admission to the CHC/SRU. At two and six months after stroke, both the RMA-A and BI-scores were significantly lower in the South African than the German sample.

### Motor and functional recovery patterns

**Figure 2:**
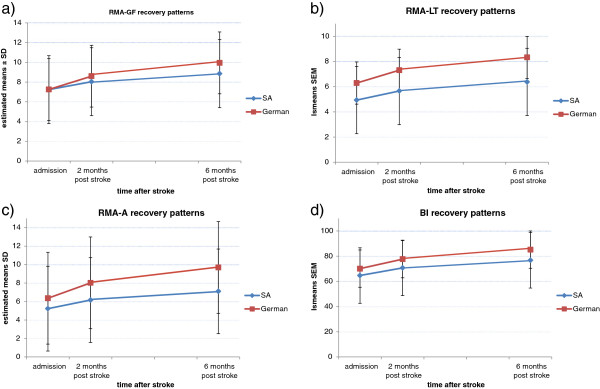
Least square means with standard error for the scores of the Rivermead Motor Assessment- (a) Gross Function, −(b) Leg and Trunk, − (c) Arm and (d) Barthel Index at onset and at two and six months after stroke for the matched South-African (SA) and German patient sample.

The results of the GLIMMIX modelling are shown in Table 3, and the least square means are visually presented in Figures 2a,b,c and d. For the RMA-GF, the interaction term ‘time*center’ was found to be significant (p = 0.006), indicating that the RMA-GF recovery slope was significantly steeper in the German than the South African sample. For the RMA-A, the significant interaction term ‘time*center’ was found to be significant (p = 0.01), indicating that the RMA-A recovery slope was significantly steeper in the German sample than the South African one. The interaction term ‘time*center’ proved not be significant in the RMA-LT (p = 0.07) and the BI-model (p = 0.35) indicating that the recovery slopes for both RMA-LT and BI did not differ significantly between patient samples.

**Table 3:** Results of the GLIMMIX modelling

	**RMA-GF**		**RMA-LT**		**RMA-A**		**BI**	
	**Estimate (SE)**	**p-value**	**Estimate (SE)**	**p-value**	**Estimate (SE)**	**p-value**	**Estimate (ES)**	**p-value**
**Center**	0.54 (0.67)	0.42	−1.05 (0.50)	0.04	−0.40 (0.83)	0.63	−3.93 (4.50)	0.38
**Time**	1.35 (0.13)	<0.0001	1.02 (0.11)	<0.0001	1.67 (0.19)	<0.0001	7.55 (1.09)	<0.0001
Center*time	−0.55 (0.20)	**0.006**	−0.30 (0.17)	0.07	−0.73 (0.28)	**0.01**	−1.51 (1.60)	0.35

RMA-GF: Rivermead Motor Assessment- Gross Function, RMA-LT: Rivermead Motor Assessment- Leg and Trunk; RMA-A: Rivermead Motor Assessment Arm Function, BI: Barthel Index, *: with.

## Discussion

In the present study, motor and functional recovery patterns were compared between stroke patients admitted to an in-patient treatment centre in Germany (developed/well-resourced) and their matched counterparts admitted to an out-patient treatment facility in South Africa (developing/under-resourced) for a six-month post stroke recovery period.

The results of the GLIMMIX showed that the recovery patterns for gross motor functioning (RMA-GF) were significantly steeper in the German patients than the South African patients. The RMA-GF has a total of 13 items that include the assessment of the ability to sit, perform transfers (lying to sitting, sitting to standing and wheelchair to chair) and walk independently. The significant differences in RMA-GF scores therefore imply that the German participants were better at performing these activities than the South African participants. Similarly, German patients demonstrated significantly faster recovery of arm function (RMA-A). These findings could be linked to differences in the process of rehabilitation associated with the typical treatment regimes in developed versus developing countries, but they do not imply a causal link.

The German centre provided in-patient treatment of high intensity, meaning that the patients received daily therapy on an average of 2 hours and 20 minutes [22]. In contrast, the South African patients received out-patient therapy at an average of once a week [18]. Thus the intensity of treatment differed significantly between the developed and developing countries, potentially impacting outcome and recovery patterns [6].

The content of physiotherapy received by the South African and German patients was similar. In both the South African [23] and the German centres [24] the most frequently practised activities were selective movements, exercises, and balance in sitting and standing. Ambulatory exercises were, however, practised less by the South African sample, which could have contributed to the improved ability of the German participants to perform these activities. Task-specific exercise is known to be effective in the rehabilitation of stroke patients [25]. The German sample received more occupational therapy (OT) than the South African participants [22]. In the study conducted at the CHCs, 99 % of the stroke patients received physiotherapy, while only 21 % received occupational therapy. The patients in the German centre engaged in domestic and activities of daily living in the OT sessions [24], activities in which the upper limb is more involved [26,27] than when performing ambulatory activities, for example [24]. The activities practised in the occupational therapy sessions are intended to contribute to upper limb recovery, which may explain the steeper RMA-A recovery curves in the German sample.

No significant differences were found in the recovery patterns for functional recovery as measured by the Barthel Index, though the German sample produced higher median scores at two and six months after stroke. This non-significant finding could be attributed to the suboptimal recovery of the South African sample with regards to basic activities of daily living as measured by the Barthel Index. With regards to the German sample the ceiling effect of the Barthel Index could have affected the recovery, the median scores of the BI of the German sample was 90 and 95 at two and six months respectively [28]. The findings relating to the recovery of the RMA-LT could not be compared, as the two groups were significantly different with regard to this outcome at baseline, which would have affected the comparison of the recovery patterns.

### Limitations of the study

As the study compared the outcomes of stroke patients from two different countries, cultural differences which are intrinsic to the patients could have affected the findings. The matching process used in the study also decreased the size of sample that could be compared. The process of matching meant that a number of participants from both settings were excluded from the study, which could have affected the findings. A major limitation is that the study used secondary data, which limited the researcher’s ability to determine what the factors were that could have influenced the recovery patterns and outcomes.

## Conclusion

The findings indicated that the German stroke population reported statistically significantly better recovery patterns for RMA-GF and RMA-A. Well-resourced rehabilitation in a German rehabilitation centre generated moregross motor and upper limb recovery when compared to less resourced outpatient services in South Africa. The findings therefore provide empirical support for perceptions held by rehabilitation professionals. The findings of this study, using secondary data, should be further investigated using prospective designs.

## Abbreviations

BI: Barthel Index; CERISE: Collaborative evaluation of rehabilitation in stroke across Europe; CHCs: Community health centres; GLIMMIX: Generalized linear mixed methods model; NIHSS: National institute of health stroke scale; RMA: Rivermead motor assessment scale; RMA-GF: Rivermead motor assessment scale gross motor function; RMA-LT: Rivermead motor assessment scale leg and trunk function; RMA-A: Rivermead motor assessment scale arm function; SA: South Africa; SRU: Stroke rehabilitation unit.

## Competing interests

The authors declare that they have no competing interests.

## Authors’ contributions

AR and LDW were the main researchers in conceptualization of the study and writing the article. MS and RM contributed to the conceptualization and finalization of the South African study, and commented on the article. WD contributed to conceptualization and finalization of both South African and European studies, and commented on the article. KP was involved in the European study and commented on the articles; he also developed the matching process used in the comparison. All authors read and approved the final manuscript.

## Pre-publication history

The pre-publication history for this paper can be accessed here:

http://www.biomedcentral.com/1472-6963/14/82/prepub
